# Optogenetics in primate cortical networks

**DOI:** 10.3389/fnana.2023.1193949

**Published:** 2023-05-22

**Authors:** Sam Merlin, Trichur Vidyasagar

**Affiliations:** ^1^Medical Science, School of Science, Western Sydney University, Campbelltown, NSW, Australia; ^2^Department of Optometry and Vision Sciences, School of Health Science, The University of Melbourne, Parkville, VIC, Australia; ^3^Florey Department of Neuroscience and Mental Health, The University of Melbourne, Parkville, VIC, Australia

**Keywords:** non-human primate, optogenetics, neural circuits, systems neuroscience, electrophysiology

## Abstract

The implementation of optogenetics in studies on non-human primates has generally proven quite difficult, but recent successes have paved the way for its rapid increase. Limitations in the genetic tractability in primates, have been somewhat overcome by implementing tailored vectors and promoters to maximize expression and specificity in primates. More recently, implantable devices, including microLED arrays, have made it possible to deliver light deeper into brain tissue, allowing targeting of deeper structures. However, the greatest limitation in applying optogenetics to the primate brain is the complex connections that exist within many neural circuits. In the past, relatively cruder methods such as cooling or pharmacological blockade have been used to examine neural circuit functions, though their limitations were well recognized. In some ways, similar shortcomings remain for optogenetics, with the ability to target a single component of complex neural circuits being the greatest challenge in applying optogenetics to systems neuroscience in primate brains. Despite this, some recent approaches combining Cre-expressing and Cre-dependent vectors have overcome some of these limitations. Here we suggest that optogenetics provides its greatest advantage to systems neuroscientists when applied as a specific tool to complement the techniques of the past, rather than necessarily replacing them.

## 1. Introduction

The functional characterization of mammalian neural systems has been greatly aided by recent technological advances. Optogenetic tools, along with chemogenetics, have been developed to selectively activate or inactivate specific neural circuits (Zemelman et al., 2002; Boyden et al., 2005; Nagel et al., 2005). Genetically tractable models have proven the power of optogenetic manipulations, with the ability to target neurons based on gene expression or time of neuronal birth and with large-scale expression of opsin channels. However, optogenetics has proven to be more difficult to apply in non-genetically tractable animal models, not least of which are the non-human primates. In this respect, genetic tractability describes the ability to readily modify an animal’s genome, as seen with knock-in or knock-out mouse models, while intractability refers to those animals that currently face barriers to genetic modifications, using current techniques.

The implementation of optogenetics to mammalian systems has led to the development of many different opsins for excitation and inhibition. Excitatory opsins, leading to cell depolarization through utilizing inward cation channels, have seen variants of channelrhodopsin (ChR2; Boyden et al., 2005) developed to provide red-shifted excitatory channels such as ChRimson (Klapoetke et al., 2014) and C1V1 (Packer et al., 2012; Prakash et al., 2012), or by increasing channel kinetics, such as ChRonos (Klapoetke et al., 2014) or ChETA (Gunaydin et al., 2010). Inhibitory opsins that hyperpolarize cells either through use of inward chloride pumps, as with halorhodopsin (HR; Gradinaru et al., 2010) and its red-shifted strain variant Jaws (Chuong et al., 2014) or outward proton pumps, as with archaerhodopsin (Arch; Chow et al., 2010), and related variant ArchT (Han et al., 2011). This vast and ever-growing library of opsins provides enough diversity in excitation spectra and channel kinetics to provide for complex combinations to maximize experimental efficiency.

The non-human primate (NHP) represents the ideal animal model for understanding neural circuit functions in humans. In many aspects of neural function, such as processing visual information, the non-human primate is the only model that realistically models human function. Therefore, in order to determine the role of homologous brain regions and their connections, experiments in NHPs are often vital. Advances in NHP optogenetics have illustrated the feasibility of using viral vectors to deliver opsin channels to specific parts of the NHP brain (Diester et al., 2011; Han et al., 2011; Tamura et al., 2012; Ruiz et al., 2013). These studies systematically addressed the numerous early technical issues in applying optogenetics to the NHP, including vector expression (Han et al., 2009; Ruiz et al., 2013) and delivery (Krauze et al., 2005; Lerchner et al., 2014; Yazdan-Shahmorad et al., 2016) and promoter choice (Lerchner et al., 2014). However, despite all the progress made in the last decade (Boyden et al., 2005; Chow et al., 2010; Han et al., 2011; Galvan et al., 2017; El-Shamayleh and Horwitz, 2019), there remain some difficulties in widespread application of optogenetics to study the NHP brain.

Here, we will discuss the advances in optogenetics in NHPs, and will outline the key considerations when designing NHP optogenetic studies.

## 2. Expression of opsins

Genetic tractability in mice has proven to be a hugely beneficial tool in targeting specific neuronal populations. Transgenic mouse lines have allowed expression of opsins in specific neurons, without the need for vector-mediated expression and its associated damage (Jaenisch and Mintz, 1974; Sharpless and Depinho, 2006). Such transgenic expression of opsins has led to exquisite functional manipulations of neurons and their connections revealing unambiguously neural circuit function of many networks in the rodent brain (Horowitz et al., 1999; Asrican et al., 2013).

In the absence of transgenic expression of opsins, the use of well-established Cre-lox expression systems (including alternatives, such as FLP-FRT) has permitted greater flexibility as well. The vast array of Cre-expressing mouse lines that are now available allows specific expression of opsins in discrete regions by targeted application of Cre-dependent vectors (Cardin et al., 2010). The flexibility of opsin used enables very precise circuit manipulation to elucidate the functional contribution of just a subset of neurons within the target region. Additionally, greater specificity of targeting has been achieved by employing intersectional targeting methods, utilizing several methods of targeting together, such as Cre/FLP double recombinase, to improve specificity (Madisen et al., 2015).

Optogenetic studies on NHPs currently lack this same level of genetic tractability, either with transgenic manipulations or with Cre-dependent targeting. That is, NHPs do not show the same ease in genome modification to express opsins, or Cre-recombinase in specific cell types, as mouse models. This is despite recent advances in CRISPR modification in primates (reviewed in Chen et al., 2016). With genetic tractability currently not being feasible in NHPs, the use of replication-deficient viral vectors has emerged as the most viable method of establishing opsin expression. Primarily, Adeno-associated virus (AAV) and lentiviral (LV) vectors have been successfully used in NHPs, with non-integrating and non-pathogenic AAV emerging as the preferred vector.

Several of the pioneering NHP studies utilized LV as an expression vector, to express ChR2 in frontal eye field (Han et al., 2009), inhibitory opsin ArchT in Areas V1 and 7a (Han et al., 2011) and ChR2 in striatum and thalamus (Galvan et al., 2012). More recently LV was also used to demonstrate the feasibility of expressing the chemogenetic channel, Hd4Mq in NHPs (Fredericks et al., 2020). Lentivirus produces somewhat higher yield of gene inserts than AAV, but the rate of expression can vary quite extensively from tissue to tissue (Han et al., 2011; Lerchner et al., 2014). Though very safe to use, it has a somewhat unfair connotation derived from its origin as a retrovirus, presenting additional biosafety concerns that may counterbalance its effectiveness.

The recent alternative to LV in NHPs has been AAV (Ohayon et al., 2013; Ruiz et al., 2013). AAV has a smaller gene of interest insert capacity and is a smaller vector facilitating spread through tissue, thereby maximizing tissue transduction (Choi et al., 2014), while producing very little immune response (Mendoza et al., 2017). This ability to transduce large areas of NHP tissue with the viral vector is vital for delivering measurable effects on brain function, since inability to transduce sufficient amount of tissue and limited delivery of light remain the two major technical limitations in using optogenetic tools in NHPs. Convection enhanced delivery improves expression (delivering larger volumes thereby forcing vector into tissue; Krauze et al., 2005; Yazdan-Shahmorad et al., 2016, 2018). This process not only delivers greater and even vector distribution (center vs. edge of vector spread), but may also increase the proportion of transduced neurons, potentially overcoming a major limitation (Yazdan-Shahmorad et al., 2016). However, the smaller cassette size means there are limitations over the amount of information each vector can possess, whereby expression of an opsin, a specific promotor and a fluorophore may reduce transduction efficiency if too large, or even exceed viable cassette size in some cases.

Adeno-associated virus serotype has also been extensively characterized in NHPs (Dodiya et al., 2010; Markakis et al., 2010; Gerits et al., 2015; Watakabe et al., 2015; Cushnie et al., 2020). These studies have revealed that AAV serotypes differ in the spread of the vector from injection site and in the number of neurons transduced (Gerits et al., 2015; Watakabe et al., 2015). While all AAV serotypes produce very good expression and spread through tissue, AAV1 and AAV5 have been shown to potentially maximize the number of neurons transduced (Gerits et al., 2015), while AAV8 and AA9 produce good spread and expression (Watakabe et al., 2015; Wang et al., 2019) and AAV2 shows reduced spread through tissue (Watakabe et al., 2015; Cushnie et al., 2020). Such is the effectiveness of AAVs to transduce NHP tissue, that almost all serotypes have been used in the application of optogenetics in the last decade.

While AAVs display excellent transduction of NHP tissue and high neuronal expression (Varenika et al., 2009), it introduces the competing need to restrict expression to intended cell types. The relatively small expression capacity of AAVs and the relatively large gene cassette size of opsin channels, leaves a little available cassette space for many larger expression promoters, somewhat limiting their use for all applications. However, this limitation can be partially overcome by exploiting known anatomical connections to provide specificity of expression.

### 2.1. Non-specific expression

The most common early approaches utilizing optogenetics in primates involved the activation or inactivation of particular brain regions to assess their functional role (Han et al., 2009). This approach has produced very interesting and reliable findings, particularly in motor circuits where changes in motor output can be readily quantified. The approach has also been used to show the role of amygdala in controlling saccades under different emotional situations (Maeda et al., 2020) and to induce forelimb movements following transduction of M1 neurons (Watanabe et al., 2020). A similar non-specific approach has also been used in sensory systems, with inactivation of IT producing reduced face discrimination (Afraz et al., 2015) and in reducing saccades following inactivation of the superior colliculus (Cavanaugh et al., 2012). This method of modulation is limited in the clarity provided over how these areas produce their actions, with excitatory and inhibitory neurons equally likely to be infected and often the glia also transfected.

### 2.2. Targeting by cell type

One of the most successful methods of selectively expressing opsins in specific subpopulations in the NHP has been through promoter-driven expression. In this case, one could argue that the use of a promoter can also act as an expression restrictor rather than expression enhancer, effectively limiting expression by use of the promoter (Rubin et al., 2020). The use of expression enhancers, such as the high-yield promoters CAG and CMV, leads to high levels of expression, but lacks selectivity, likely producing gene product in excitatory and inhibitory neurons and perhaps also in glia. Instead, when using promoters designed as restrictors, such as for tyrosine hydroxylase (TH), expression will be limited to TH expressing cells, greatly reducing the overall number of expressing neurons. This tradeoff in expressing neurons can be balanced with viral load to ensure sufficient expression is achieved to produce meaningful results. This approach has been successfully used to show that activation of dopaminergic TH neurons in the NHP ventral tegmental area produces reward associated behaviors (Stauffer et al., 2016). Similar approaches have been used to selectively transduce Purkinje cells in the NHP cerebellum using an L7 promoter (El-Shamayleh et al., 2017), koniocellular neurons in the lateral geniculate nucleus using a CaMKIIa promoter (Klein et al., 2016) and GABAergic neurons in primary visual cortex using a Dlx5/6 promoter (Dimidschstein et al., 2016; De et al., 2020).

CaMKIIa promoters selectively target expression in excitatory neurons in primary visual cortex (Nassi et al., 2015; Chernov et al., 2018; Ju et al., 2018), primary motor cortex (Ebina et al., 2019) and temporal cortex (Tamura et al., 2017), while still attaining excellent enhancement of opsin expression (Lerchner et al., 2014). CaMKIIa promoter has also been shown to allow selective transduction of interhemispheric connections in NHP visual cortex (Nakamichi et al., 2019). Similarly, hSyn promoters have led to selective neuronal (though not selectively excitatory) expression of inhibitory opsin Jaws in FEF, reducing and delaying visual saccade (Acker et al., 2016) and in MT to transiently suppressing direction discrimination (Fetsch et al., 2018).

### 2.3. Targeting by connectivity

Perhaps the most effective method of selectively transducing functional networks in the NHP is to target the known connectivity of projection neurons. At its simplest, this could involve injection of an expression vector at the soma or terminals of a projection and stimulation at the other (Inoue et al., 2015; O’Shea et al., 2018). Hence, only neurons projecting from one region to another can possibly be both transduced and optically stimulated. This powerful approach has been used to show that motor corticothalamic projections are modulatory (Galvan et al., 2016), excitation of FEF to SC projection elicits visual saccade (Inoue et al., 2015), stimulation of visuomotor basal ganglia circuit produces contralateral saccade (Amita et al., 2020) and to interrogate frontoparietal and frontooccipital connections (Fortuna et al., 2020). This approach, while exclusively targeting connections between areas, it is limited by the directional specificity of the delivery vector, with both AAV and LV displaying bidirectional transduction.

Specificity can be improved upon by the combination of vectors to bidirectionally transduce a connection. This would involve the combination of largely anterograde AAV infection at the target neuron somata and largely retrograde AAV or CAV2 injection at the target neuron axon terminals to limit expression to a projection while avoiding any reciprocal connections. If this is also employed along with Cre-dependent and Cre-expressing recombination of vectors at somata and terminals, respectively, even greater specificity can be achieved by ensuring only neurons that take up both vectors will express the desired opsin (Nurminen et al., 2018). This method unambiguously labels a unidirectional connection within a functional network and allows specific activation or inactivation without affecting a reciprocal connection. This method has been used to functionally characterize the role of V2 to V1 feedback in modulating receptive field size and response gain (Nurminen et al., 2018). It is important to note that limiting one of the vector injection volumes increases the likelihood of restricting transduction to unidirectional connections, which may reduce the numbers of transduced neurons.

## 3. Delivering optical stimulation

### 3.1. Surface illumination

Delivery of sufficient light, especially to deeper structures remains one of the key technical difficulties in NHP optogenetics. The general popularity of the excitatory opsin ChR2 is apparent, not only in primate optogenetics, but also in other animal systems (Gerits et al., 2012; Tamura et al., 2012; Ruiz et al., 2013). ChR2 is strongly activated by blue light (473 nm peak activation), meaning that it primarily responds to lower wavelength, but high energy light. This means that particularly for ChR2, the ideal activation wavelength produces increased tissue diffraction and poor tissue penetration (Abaya et al., 2014). The commonly used inhibitory opsins Arch (or ArchT) and HR, with peak activation wavelengths of 532 and 560 nm, respectively (Chow et al., 2010; Han et al., 2011), are also not exempt from the same limitations of light penetration through tissue.

**FIGURE 1:**
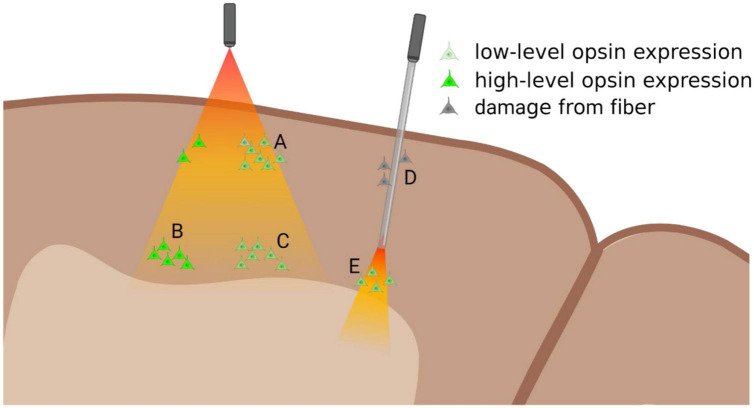
Diagram of relationship between opsin expression and light-delivery. Surface illumination (left) will deliver sufficient light to superficial layers, where even slightly lower levels of opsin expression can lead to successful modulation **(A)**. Light intensity dissipates in deeper layers where optogenetic modulation requires high-levels of opsin expression **(B)**, while low-levels will not be modulated **(C)**. Multi-photon light activation could be used to access these deeper layers, though the area of light-activation will be greatly reduced. Implantable optic fibers (right) can be used to deliver light to deeper structures **(E)**, though they might damage superficial layers **(D)** and could potentially disrupt functional column activity. Figure created with Biorender.com.

The development of red-shifted and also far-red-shifted optogenetic channels has permitted greater tissue penetration, potentially accessing deeper structures. ChR2 variants, such as ChRimson and C1V1, have been employed to normalize responses in V1 (Nassi et al., 2015), produce sustained gamma oscillations in M1 (Lu et al., 2015), show that corticothalamic afferents are modulatory (Galvan et al., 2016), produce a fast saccade following V1 stimulation, though not resembling visual stimulation (Ju et al., 2018) and to show neural adaptation in IT (Fabbrini et al., 2019). These studies were made possible by the greater penetration into cortical layers afforded by the higher wavelength light (Figure 1).

However, even in cortical tissue light still penetrates robustly only to about layer 4 or 5, making accessing deeper subcortical structures without optical guides impossible. Increasing power to provide further penetration of light, leads to light-induced heating of superficial layers, pushing superficial layers toward threshold, thereby introducing aberrant firing within the cortical column (Nurminen et al., 2018). Inhibition studies are particularly susceptible to heating artifacts, since excitation from heat artifacts is likely to interfere with or overcome the functional inactivation. As most cortical areas are arranged in functional columns through the cortical depth, inactivation at any stage of the column, while also creating heat-induced activation can potentially invalidate findings of the study. As such, even successful inactivation of target neurons within a functional column, may be susceptible to heat-induced activation of output targets of the inactivated neurons, negating the manipulation of the neural circuit. While optogenetic activation studies are open to similar artifacts, it could be argued that while the non-specific heat-activation of circuit components may cloud the role of the targeted neurons, there is less likelihood of obscuring the function of the entire neural circuit.

Multi-photon targeting of opsins allows for greater penetration of higher-wavelength light, potentially reaching deeper targets (Chaigneau et al., 2016; Forli et al., 2018; Adesnik and Abdeladim, 2021), but only activating opsins at the specific site of light beam convergence. This approach is potentially highly useful in probing functional columns or microcircuits (Adesnik and Abdeladim, 2021), but may not generate sufficient activation to evoke response changes in interareal neural circuits.

### 3.2. Implantable light-guide

Fiber optic implants allow light to deeper cortical layers or subcortical targets (Figure 1), generally employing a 100–200 μm diameter flat-ended optical implant (Dubois et al., 2018). Implantation of these devices damages superficial layers, potentially disrupting both apical dendrites of deeper layers, and hence interfering with the canonical cortical column, potentially nullifying the results of these studies of cortical function (Figure 1). Alternatively, optical implants can be designed to replicate electrodes, with tapered tips, that reduce tissue damage upon insertion (Boutte et al., 2017). However, with the reduction in tissue damage, there is greater scattering of light, due to the tip angle, necessitating greater light intensity, and risking damage to the surface through spill of aberrant light outside the implanted light guide.

Self-contained optical devices are being developed with inbuilt microLED arrays and at times penetrating optical guides (McAlinden et al., 2019; Mondello et al., 2021). These devices would allow for selective power increase via the optrode shank, without increase in light power to the cortical surface, while also positioning microLEDs in direct contact with optrode shanks, which minimizes light loss. This approach is currently limited by microLED power thresholds, but with advances in this area occurring rapidly, these devices might represent the ideal method of light delivery in NHP (reviewed in Hee Lee et al., 2022).

## 4. Isolating function in a network

**FIGURE 2:**
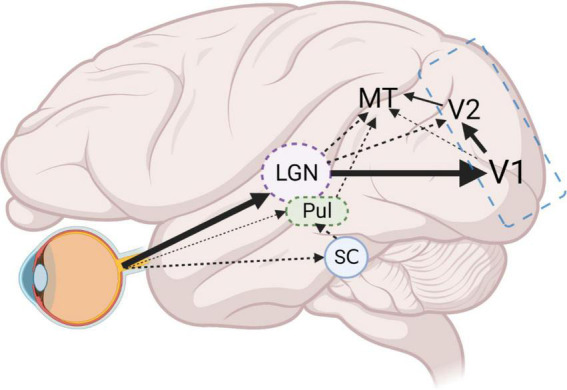
Schematic of example LGN-V1-MT circuit. Dense projections from the retina, to LGN and in turn to V1, form the main visual input pathway. From V1, the densest dorsal stream projections reach MT via V2. However, a smaller projection from V1 (layer 4B) directly to MT, also exists. Furthermore, direct projections from LGN to MT, and LGN to V2 then to MT, bypass V1 entirely. An additional retinal output to pulvinar nucleus (Pul) and the stronger connections to the Pul via the superior colliculus (SC) also project directly to MT. Optogenetic probing of the LGN to MT projection requires simultaneous silencing of alternate projections from V1, V2, and Pul. High magnification factors in V1 and V2 in comparison to MT, mean large areas of tissue will need to be illuminated to silence these projections, making optogenetic silencing infeasible. Employing classical cooling of V1 and V2 (blue outline) will effectively remove V1 and V2 related circuits, allowing for the manageable modulation of remaining direct MT projections. Figure created with Biorender.com.

Perhaps the greatest difficulty in applying optogenetics to primate cortical networks is the complexity of interconnected cortical areas. While this complex connectivity is not a feature of NHPs alone (Markov et al., 2013, 2014; Oh et al., 2014; Bota et al., 2015; Gamanut et al., 2018), when combined with the previously mentioned limitations of applying optogenetics to NHPs, it represents a unique challenge. For instance, to use optogenetics to probe the function of the direct LGN to MT projection, suggested to underlie the phenomenon of blindsight (Morland et al., 2004; Rees, 2008), one could design a study to specifically express an inhibitory opsin in the LGN to MT neurons using an anterograde Cre-dependent and retrograde Cre-expressing approach. This would ensure that only LGN to MT neurons express opsins, permitting precise loss of function experimentation. However, LGN also projects in a major way to V1, which projects to MT directly and indirectly via V2. Significantly, a small projection from koniocellular layers of LGN to V2 also exists (Hendry and Reid, 2000) bypassing V1 and whether these are branches of the same MT-projecting neurons is unknown. In this case, silencing of the slow-transduction koniocellular pathway to MT, but not the intact V2 projection, or the intact, fast-transduction magnocellular pathway via V1 may all complicate interpretation of results (Figure 2).

We suggest that it is vital that optogenetics be employed as a powerful tool to compliment traditional cooling and pharmacological blockade techniques, such as cooling of V1 to study the direct and indirect projections from LGN to MT (Jayakumar et al., 2013). Cooling or GABAergic blockade of V1/V2 combined with time-locked laser-induced silencing of LGN to MT pathway would provide unambiguous functional characterization of the direct MT projection. As such, optogenetics in primates should be viewed as a powerful tool when used in conjunction with established techniques, but could produce ambiguous findings when applied alone. It is therefore vital that established research groups employ optogenetics to facilitate their existing methods, rather than replacing them.

This approach is necessary, as, in our example, the numerous avenues of connectivity between LGN and MT would create confounds if optogenetics were employed alone. This complexity of connections would provide numerous avenues for MT activation, complicating an all optogenetic approach. In addition, differences in magnification factors across hierarchical sensory processing areas means that simultaneous optogenetic inactivation of multiple areas at once becomes harder and harder to achieve by optogenetics alone. As such, employing optogenetics to try to specifically modulate direct LGN to MT connections, while simultaneously cooling V1 and V2 would provide a more reliable means of specific optogenetic probing of this direct pathway. Additionally, direct retinofugal connections via the pulvinar nuclei to MT (Warner et al., 2010, 2012) and via the superior colliculus to MT (Berman and Wurtz, 2008) further complicate this circuit, though it is unclear if anesthesia differentially affects LGN and pulvinar activity (Figure 2).

This pattern of overlapping cortical connections is not specific to the MT, and must be a consideration for planning optogenetic approaches. As such, we strongly advocate for the inclusion of optogenetics in conjunction with existing approaches to probe complex neural circuits. This does not mean that all-optical approaches do not have a purpose (Hochbaum et al., 2014), but perhaps represent an ideal method of probing activity in a particular functional domain, rather than complex interareal neural circuits.

## 5. Conclusion

The adaptation of optogenetics to primates is a research area that is rapidly growing. Recent advances in technical features of viral vector delivery, opsin sensitivities and dynamics and light delivery devices have aided in making optogenetic manipulations of primate neural networks highly feasible—to the point of triggering the development of optogenetic implants for human trials (Zhang et al., 2022). These advances will likely continue in the future, providing a toolkit for opsin-based interventions in both biomedical research and treatment.

One aspect that must bear consideration is not open to the same technological advances. It is the ability to isolate a target neural network for manipulation. The complexity of both human and non-human primate neural networks represents a constant challenge for both functional studies and therapeutic application of optogenetics. The ability to specifically manipulate a neural network optogenetically, without simultaneously altering other interconnected network functions, is unlikely to be improved through short-term technological advances and remains the greatest limitation on its widespread use in non-human primates. However, depending upon the question being studied and the pathways involved, appropriate controls may be adopted to overcome many of these limitations.

## Data availability statement

The original contributions presented in this study are included in the article/supplementary material, further inquiries can be directed to the corresponding author.

## Author contributions

SM and TV wrote the first draft of the manuscript. Both authors contributed to the article and approved the submitted version.
